# Population Genetics of *Haliotis discus hannai* in China Inferred Through EST-SSR Markers

**DOI:** 10.3390/genes16010073

**Published:** 2025-01-10

**Authors:** Hongsu Yang, Zhou Wu, Guangyu Ge, Xiujun Sun, Biao Wu, Zhihong Liu, Tao Yu, Yanxin Zheng, Liqing Zhou

**Affiliations:** 1 State Key Laboratory of Mariculture Biobreeding and Sustainable Goods, Yellow Sea Fisheries Research Institute, Chinese Academy of Fishery Sciences, Qingdao 266071, China; m17865567520@163.com (H.Y.);; 2 Fisheries College, Zhejiang Ocean University, Zhoushan 316022, China; 3 Changdao Enhancement and Experiment Station, Chinese Academy of Fishery Sciences, Yantai 265800, Chinazhengyanxin1989@163.com (Y.Z.)

**Keywords:** *Haliotis discus hannai*, EST-SSR development, genetic variation

## Abstract

Background/Objectives: The Pacific abalone *Haliotis discus hannai* originated in cold waters and is an economically important aquaculture shellfish in China. Our goal was to clarify the current status of the genetic structure of Pacific abalone in China. Methods: In this study, eighteen polymorphic EST-SSR loci were successfully developed based on the hemolymph transcriptome data of Pacific abalone, and thirteen highly polymorphic EST-SSR loci were selected for the genetic variation analysis of the six populations collected. Results: The results showed that the average number of observed alleles was 8.0769 (RC)-11.3848 (DQ) in each population. The number of observed alleles in the DQ, NH, and TJ populations was significantly higher than that in the RC population. The cultivated population outside the Changshan Islands has experienced a 22.79% reduction in allele diversity compared to the wild population of DQ. The pairwise F_st_ values and analysis of molecular variance (AMOVA) revealed significant population differentiation among all populations except DQ and NH populations, with RC and ZZ cultured populations exhibiting the largest population differentiation (F_st_ = 0.1334). The phylogenetic tree and structural analysis divided the six populations into two groups (group 1: NH, DQ, and ZZ; group 2: DL, TJ, and RC), and there was no relationship between geographical distance and genetic distance. Conclusions: These results may reflect the large-scale culture from different populations in China and the exchange of juveniles between hatcheries. Different breeding conditions have led to a higher degree of genetic differentiation between the RC and ZZ populations. This study enables a better understanding of the genetic diversity and structure of current Pacific abalone populations.

## 1. Introduction

The Pacific abalone *Haliotis discus hannai* is a cold-water marine shellfish with high economic and nutritional value that is naturally distributed in Japan, South Korea, and the coasts of the Shandong Peninsula and Liaodong Peninsula in northern China. However, due to overfishing, the wild resources of Pacific abalone in China suffered serious damage and were almost exhausted by the 1970s. Since the late 1980s, the large-scale culture of Pacific abalone has been carried out in China. Subsequently, with the continuous development of breeding technology and scale, the focus of Pacific abalone culture has gradually shifted to the southern coastal areas of China. Currently, Pacific abalone is the main cultured abalone species in China [1,2].

In this case, Pacific abalone was transported to the south for culturing before winter and then transported back to the north before the following summer [3], and frequent exchanges of juveniles occurred between the hatcheries [4]. These phenomena have removed the geographic barriers of Pacific abalone populations in different regions and increased the genetic exchanges among them, which may lead to a decrease in genetic variation, thereby affecting the genetic structure of populations. One of the important tools for fisheries management and species conservation is monitoring populations’ genetic variation and structure [5,6]. Therefore, it is necessary to analyze the genetic variation in different abalone populations in China.

Microsatellites or simple sequence repeats (SSRs), which are tandem repeats of short (1–6 bp long) sequences [7,8], have many ideal traits, such as high polymorphism, high variability, codominance, specificity under polymerase chain reaction (PCR) amplification, and easy analysis of results. These markers have been widely used in population genetics research on fish [9], shrimp [10], shellfish [11,12,13], and others. SSRs include genomic SSRs and expressed sequence tag SSRs (EST-SSRs). Relatively speaking, developing SSRs from genomic libraries is laborious and costly [14]. EST-SSRs are derived from cDNA libraries with a high proportion of functional annotation information and may be closely related to genes controlling important genetic characteristics [15]. These markers have high versatility in closely related species [14,16,17,18] and can also help identify candidate functional genes and improve the efficiency of marker-assisted selection (MAS) [19,20,21]. With the rapid development of next-generation sequencing technology, economical and rapid high-throughput sequencing has become possible [22]. Transcriptome sequencing has played an important role in studying non-model species without reference genome information [23], as it greatly reduces the dependence on genomic SSRs. In recent years, many scholars have successfully developed SSR markers for economically important aquatic organisms based on transcriptomes. For example, Li et al. (2015) successfully screened 36 polymorphic EST-SSR markers based on the ESTs of *Paramisgurnus dabryanus*, and these markers laid a foundation for future population genetic research and large-scale breeding [24]. Ge et al. (2017) isolated 14 polymorphic EST-SSR markers from *Verasper variegatus* transcriptome data [25]. Chen et al. (2016) developed 36 polymorphic EST-SSR markers from ESTs of *Paphia textile* [12]. Wang et al. (2021) obtained five polymorphic EST-SSR markers by screening transcriptome data from the ark shell (*Scapharca broughtonii*) [26]. These markers were applied to the genetic analysis of four different geographic populations to explore the impact of the introduction of the Korean ark shell on the genetic diversity of the native population in China.

Our goal was to elucidate the current status of the genetic structure of different populations of Pacific abalone in China. EST-SSRs were developed from transcriptome data and used to analyze the genetic structure of six populations. The results of this study will help enrich the EST-SSR molecular markers of Pacific abalone, elucidate the status of the genetic structure of the populations of Pacific abalone in China, and provide valuable information for the protection of Pacific abalone germplasm resources.

## 2. Materials and Methods

### 2.1. Animals and Sample Collection

**Figure 1:**
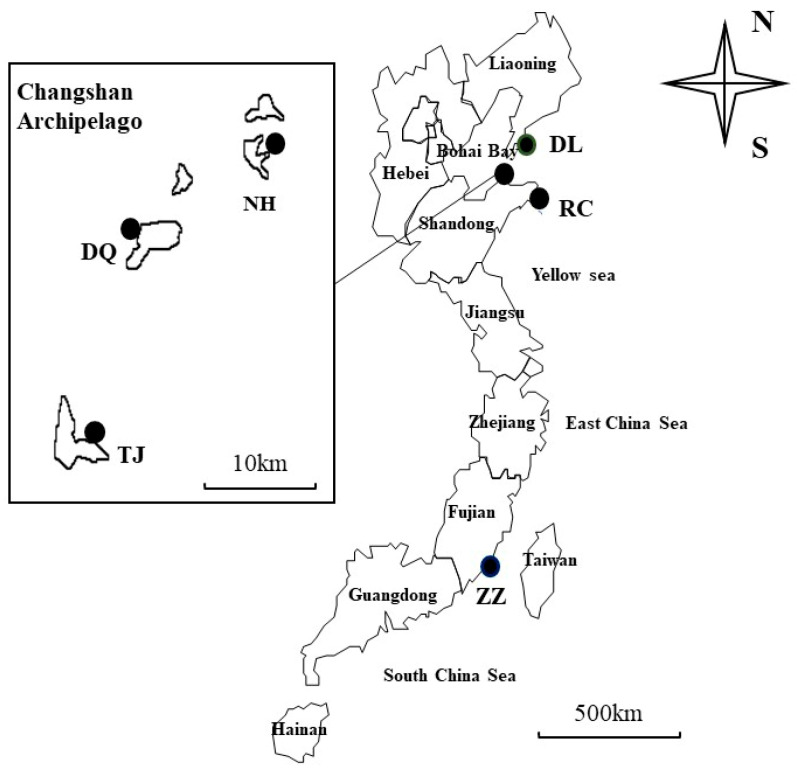
The sample source of Pacific abalone.

Six populations of Pacific abalone were collected from Zhangzhou in Fujian Province (ZZ: 44 individuals), Dalian in Liaoning Province (DL: 48 individuals), Rongcheng in Shandong Province (RC: 39 individuals), and the Changshan Archipelago, which included Nanhuangcheng Island (NH: 35 individuals), Daqin Island (DQ: 39 individuals), and Tuoji Island (TJ: 49 individuals), from August 2020 to March 2021 (Table 1, Figure 1). The ZZ population has been cultivated in Zhangzhou for a long time and used as a breeding and culture population for generations. The DL population is the overwintering population bred from Dalian to Penglai. The RC population has been a breeding and culture population for generations. The NH population has been a breeding and culture population for generations and uses the bottom sowing culture breeding mode. The breeding mode of the TJ population is raft cultivation, and it has been a breeding and culture population for generations. The DQ population is a wild population. The foot tissues were collected and placed in cryopreservation tubes for quick freezing in liquid nitrogen and stored in an ultralow-temperature refrigerator at −80 °C for subsequent DNA extraction.

### 2.2. Mining and Primer Design of EST-SSRs

EST-SSR markers were screened from the Pacific abalone hemolymph transcriptome data. MISA v2.1 software (default parameters, http://pgrc.ipk-gatersleben.de/misa/, accessed on 6 January 2025) was used to identify and locate the EST-SSRs and determine the type and frequency of microsatellites according to the repeat sequence size. Primers flanking EST-SSRs were designed using Primer 5.0 software. The major primer design parameters were melting temperature (approximately 60 °C), CG content (40–60%), primer size (20–30 bp), and product size (100–280 bp).

### 2.3. Screening EST-SSRs for Polymorphism

A total of 308 EST-SSRs (the number of dinucleotide to pentanucleotide repeat SSRs was 77) identified from the transcriptome were randomly selected for further polymorphism screening. All primers were synthesized by Beijing Tsingke Biotechnology Co., Ltd. Qingdao Branch (Qingdao, China). Genomic DNA was extracted from Pacific Abalone foot tissue using traditional proteinase K and phenol–chloroform extraction protocols and diluted to a concentration of 50 ng/μL [27]. Screening of SSRs was performed in two steps. In the first step, the preliminary SSR screening was validated by polymerase chain reaction (PCR) and 1% agarose gel electrophoresis of samples from five Pacific abalones. PCR amplification was performed in a 20 μL reaction volume containing 10 μL of 2× Taq Plus Master Mix II (Vazyme, Nanjing, China), with 0.8 μL of each primer at 10 μM, 1 μL of 50 ng/μL genomic DNA, and 7.4 μL of ddH_2_O. The PCR conditions were as follows: 95 °C for 3 min followed by 30 cycles of 95 °C for 15 s, 30 s at the locus-specific annealing temperature, and 72 °C for 1 min, with a final elongation step at 72 °C for 5 min. In the second step, after selecting the stably amplified SSR primers for fluorescence modification, PCR amplification was performed again on the template DNA of 18 samples (3 samples for each population) with the same amplification system and conditions as those in the first step. After a molecular marker was added, the ABI 3730 Genetic Analyzer was used to perform capillary electrophoresis genotyping. Gene Mapper 4.1 software was used for data analysis to screen polymorphic EST-SSR markers.

### 2.4. Application of EST-SSRs

The screened polymorphic primers were used for analyzing the genetic variation in Pacific abalone in this study. The forward primer from each pair was 5′-end labeled with 6-FAM, ROX, TAMRA, or HEX dyes, and PCR amplification (the same as in 2.3) was performed on the DNA template of all samples. The amplified PCR products from the same DNA template were combined with three pairs of different fluorescently labeled primers. The mixed PCR products were sent to Beijing Tsingke Biotechnology Co., Ltd., Qingdao Branch, for genotyping.

### 2.5. Data Analysis

The number of alleles (Na), effective number of alleles (Ne), observed heterozygosity (Ho), expected heterozygosity (He), inbreeding coefficient (F_is_), Hardy–Weinberg equilibrium (HWE), polymorphic information content (PIC), and genetic differentiation coefficient (F_st_) were obtained by using Popgene version 1.32 and GenAlEx 6.501 software. The differences in genetic diversity between populations were analyzed by using the Kruskal–Wallis test in SPSS 25 software. The frequencies of null alleles across all populations (F null) were estimated using Genepop version 4.7.5 software. The genetic distance between populations based on Nei’s standard was calculated, and then the phylogenetic tree was constructed by using the Neighbor-Joining method with Mega version 11 software. An analysis of molecular variance (AMOVA) was calculated using GenALEx 6.503 software and was used to distinguish genetic variation between populations. STRUCTURE version 2.3.4 software was used to analyze the genetic structure of the Pacific abalone populations based on Bayesian inference. The following conditions were set by the Markov chain Monte Carlo (MCMC) method (100,000 repetitions per cycle, K = 1–15), and ten independent runs were undertaken for each value of K to verify the consistency of the results. Finally, using the Structure Harvester (http://taylor0.biology.ucla.edu/structureHarvester/, accessed on 13 April 2022) website, the most suitable K value was obtained by calculation [28]. We calculated the correlation coefficient between geographic distance and genetic distance among populations using the R package vegan [29].

## 3. Results

### 3.1. Mining for EST-SSRs from Pacific Abalone Hemolymph Tissue Transcriptome Data

A total of 80,032 unigenes were obtained from transcriptome sequencing data and were used for the mining of EST-SSRs. A total of 415,996 potential SSRs were identified by using the MISA tool (Table 2). The number of SSR-containing sequences was 36,665. The number of sequences containing more than one SSR was 22,605. The most common type was dinucleotide repeats (160,881, 38.67%), followed by tetranucleotide repeats (100,776, 24.23%) and mononucleotide repeats (82,256, 19.77%). It is clear that dinucleotide SSRs are the dominant type.

### 3.2. Validation and Development of Polymorphic EST-SSR Markers

The 308 pairs of primers were used for the PCR amplification of genomic DNA from five individuals. After detection by agarose gel electrophoresis, it was found that 83 pairs of primers could amplify bright target bands, and the amplified product reached the expected size. The 83 pairs of primers were subjected to polymorphism screening, and a total of 18 pairs of polymorphic primers were finally obtained. Table 3 shows the basic information and fluorescence modification of primers for 13 polymorphic EST-SSR markers that were successfully developed.

### 3.3. Genetic Variability of Different Populations

Thirteen successfully developed polymorphic EST-SSR loci were selected for genetic variation analysis in six Pacific abalone populations. The genetic variation indices of the six Pacific abalone populations are shown in Table 4. All thirteen microsatellite loci were polymorphic in the examined populations (PIC > 0.5), and the PIC value ranged from 0.5667 to 0.8475 with a mean of 0.865. A total of 774 alleles were detected in 254 individuals. The average Na of each locus ranged from 8.0746 in the RC population to 11.3848 in the DQ population. The average Ne of each locus ranged from 4.7590 in the ZZ population to 5.8871 in the TJ population. The average Ho and He of each locus were 0.7135 (NH)-0.7787 (DL) and 0.7605 (ZZ)-0.8299 (TJ), respectively. The average Na was 11.3846, 10.7692, and 11 in the DQ, NH, and TJ populations, respectively, and 8.0769 in the RC population, showing that the DQ, NH, and TJ populations were significantly more variable than the RC population (Kruskal–Wallis test, H = 14.755, df = 5, *p* = 0.011). There was no significant difference in the average *He* among the six populations (Kruskal–Wallis test, H = 3.358, df = 5, *p* = 0.645). According to the Hardy–Weinberg equilibrium (HWE) test, among 78 population–locus pairs (6 populations × 13 loci), 59 were at HWE (*p >* 0.004, after Bonferroni correction), and 19 deviated from HWE (*p <* 0.004). The F_is_ values of the 19 pairs that deviated from the HWE were all positive. The frequencies of null alleles estimated across all populations ranged from 0.0000 and 0.3135 (Table 4). Among these, 27 population–locus pairs (35%) showed no evidence of null alleles. The incidence of null alleles was generally low, with most population–locus pairs exhibiting a low frequency of null alleles (91% of population–locus pairs had F null *<* 0.20).

### 3.4. Population Genetic Structure

**Figure 2:**
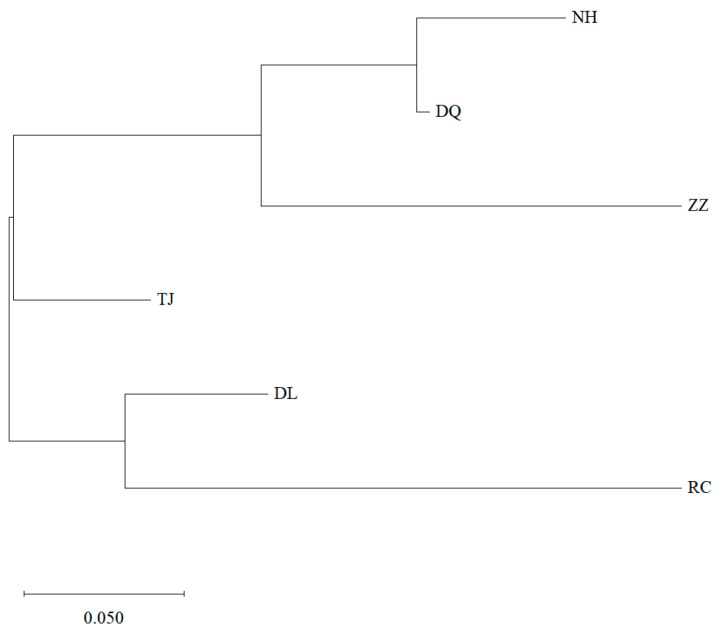
Dendrogram generated from Neighbor-Joining method cluster analysis of six populations of *H. discus hannai*.

**Figure 3:**
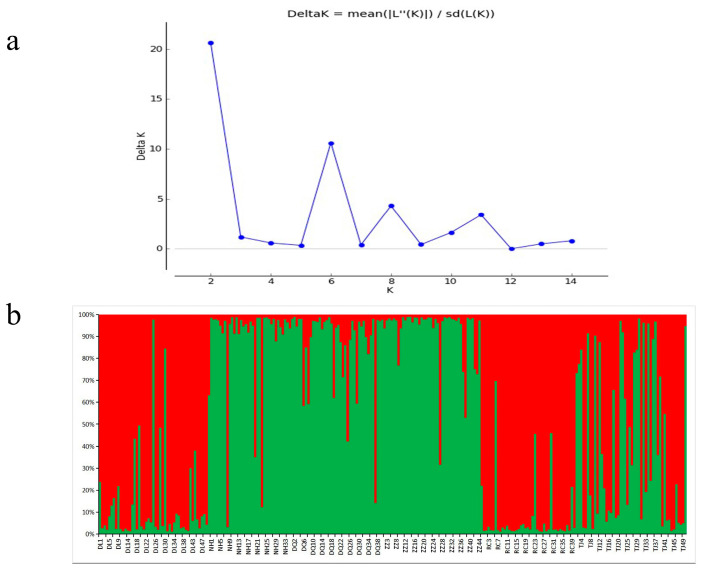
(**a**) STRUCTURE analysis of the rate of change in ΔK between consecutive K values; (**b**) Bayesian inference of the population structure of 254 individuals from six Pacific abalone populations at K = 2, as determined by STRUCTURE. Each of the two colors represents a different genetic cluster. Each line represents 1 individual.

The genetic distance between populations and the F_st_ value of genetic differentiation are shown in Table 5. The genetic distance and the genetic differentiation between populations ranged from 0.0511 to 0.4167 and from 0 to 0.1334, among which the genetic distance and genetic differentiation between the ZZ and RC populations were the largest (0.4167, 0.1334), and the genetic distance and genetic differentiation between the DQ and NH populations was the smallest (0.0511, 0). The AMOVA tests (Table 6) showed that when the six Pacific abalone populations were clustered into one group, 4% of the variation was among populations, 9% of the variation was among individuals, and 87% of the variation was within individuals, with the genetic differentiation among populations reaching a significant level (*p* = 0.001); When the six Pacific abalone populations were divided into the wild group and cultured group, 1% of the variation occurred among groups, 12% among individuals, and 87% within individuals, with the genetic differentiation between groups reaching a significant level (*p* = 0.001) The phylogenetic tree (Figure 2) clearly showed that the six populations were grouped into two major branches. DL and RC clustered together to form a large branch, and NH, DQ, and ZZ clustered together and then clustered with TJ to form another large branch. In simulations of the Bayesian clustering method with STRUCTURE 2.0 software, the ΔK value clearly suggested two clusters as the most likely population structure (Figure 3). The structural analysis (Figure 3) divided the six populations into two groups (group 1: NH, DQ, ZZ, and TJ; group 2: DL and RC), which was consistent with the phylogenetic tree. It is obvious that some individuals were admixed across populations. The Mantel test revealed an r-value of 0.3494 for the Mantel statistic, with a *p*-value greater than 0.05 (*p* = 0.174), indicating no significant correlation between geographical distance and genetic distance among the populations.

## 4. Discussion

### 4.1. Population Genetic Variation Analysis

Microsatellite markers can be used not only to analyze the genetic variation in populations but also to construct genetic linkage maps of species, perform quantitative trait locus (QTL) mapping [30,31,32,33,34], and facilitate the development of marker-assisted selection (MAS) in the genetic breeding of aquatic organisms [21,35,36]. In this study, 308 EST-SSR loci were isolated from transcriptome data, and primers were designed for PCR amplification. Then, the polymorphism of primers was detected by capillary electrophoresis combined with fluorescence modification, and the EST-SSR loci with high polymorphism were screened. Compared to the screening of polymorphic SSR loci by silver staining, the use of fluorescence has many advantages, including simple experimental operation; automatic identification and collection of data through software to improve efficiency; and the ability to identify alleles that are only 1–2 bp apart to improve data accuracy [37,38]. PIC is an important indicator for measuring the polymorphism information of SSR loci [26]. Each EST-SSR locus selected in this study had high polymorphism (PIC > 0.5). The 19 tests’ results deviated significantly from *HWE* (*p* < 0.004), and the inbreeding coefficient F_is_ was positive (range from 0.0215 to 0.6073). It is inferred that the reason for the significant deviation from HWE is heterozygote deficiency [10]. The breeding population may be prone to nonrandom mating, artificial or natural selection during the breeding process, and the occurrence of inbreeding, which may lead to heterozygote deficiency. Moreover, the genetic drift in DQ wild population leads to heterozygote deficiency. These phenomena have also been reported in other shellfish, such as pacific oysters (*Crassostrea gigas*) [39] and hard clams (*Meretrix meretrix*) [40]. When the null allele frequency was low (F null < 0.20), it usually had less effect on the genetic analysis results. In this study, most population–locus pairs had a low frequency of null alleles. In addition, EST-SSR markers exhibit lower null allele frequencies than genomic SSR markers, thus decreasing the effect of null alleles on the results of population genetic analysis [41]. Furthermore, their high transferability makes them applicable across different species [42,43]. In conclusion, the 13 EST-SSR loci screened here are ideal loci for population genetic analysis.

In this study, the average Na and He of the five cultured Pacific abalone populations was 9.6308 (range from 8.0749 to 11.3846) and 0.8009 (range from 0.7605 to 0.8299), respectively, and the average values of Na and He in the wild population were 11.3846 and 0.8055, respectively. In general, each population has a high level of genetic diversity. Among them, the average Na and Ne values of the cultured populations in the present study were slightly higher than those of Chen (2016) and Li (2007). Chen et al. (2016) used 14 pairs of microsatellite markers to study fifteen cultured Pacific abalone populations in northern and southern China and found that the mean Na and He per population was 7.552 (4.857–9.500) and 0.6501 (0.549–0.742), respectively [44]. Li et al. (2007) observed that the mean Na and He of five cultured Pacific abalone populations in northern China was 8.7 (8.0–9.4) and 0.774 (0.754–0.787), respectively [4]. The Na and Ne of the DQ wild population in the present study was similar to the Na (12.8, 11.7–14.2) and Ne (7.928, 0.761–0.846) values of the ten wild abalone populations in Japan and South Korea surveyed by Park et al. (2012) [45]. In this study, the Na values of the DQ, NH, and TJ populations located in the Changshan Archipelago were significantly higher than those of the RC population. The Changshan Archipelago is one of the original areas where Pacific abalone was found in China. According to the Regulations on the Management of Aquatic Organism Proliferation and Release put forward by the government, since May 2009, the proliferation and release of exotic or hybrid varieties of Pacific abalone in the Changshan Archipelago natural sea area has been prohibited, and we believe that the wild resources of Pacific abalone have been restored to a certain extent. The resource management measure of Pacific abalone in the region may be the main reason for the high levels of Na and Ne values of DQ, NH, and TJ populations. If NH and TJ are excluded from the cultured populations of the Changshan Archipelago, then the mean Na of the cultured populations is 8.79 (6–14), which is similar to the results observed by Chen et al. (2016) and Li et al. (2007) [4,44]. Compared with the wild DQ population, the Na of the cultured populations decreased by 22.79%, which was also similar to the results of Chen et al. (2016) [44]. In a previous study by Li et al. (2007) [4], a 76% reduction in the number of alleles in hatchery populations was observed. Decreased genetic diversity was found using microsatellites not only in Pacific abalone but also in aquatic species such as black carp (*Mylopharyngodon piceus*) [46], Manila clam (*Ruditapes philippinarum*) [13], and giant grouper (*Epinephelus lanceolatus*) [47]. Relative to wild populations, reduced genetic diversity appears to have become a feature of cultured populations, possibly due to the founder effect during domestication [47]. In addition, natural and artificial selection in the culture environment may affect the allelic composition of hatchery populations [4,48,49].

### 4.2. Population Genetic Structure Analysis

The fixation index (F_st_) between populations is an important value to measure the genetic differentiation of each population. An F_st_ value of 0–0.05 indicates low differentiation, while a value of 0.05–0.15 indicates moderate differentiation. The majority of pairwise F_st_ values of the six populations in the present study were greater than 0.05 and less than 0.15, indicating that there was moderate genetic differentiation among them, and no high degree of genetic differentiation between the southern population (ZZ) and the northern populations. The AMOVA test showed that the genetic variation mainly came from intrapopulation variation. Moreover, it is clear that six populations were divided into two groups by phylogenetic tree and structural analysis. The NH, DQ, ZZ, and TJ populations were clustered together, and the DL and RC populations were clustered into another group. There was no significant correlation between geographical distance and genetic distance in our result (r = 0.349, *p* > 0.05); we speculate that the mass-scale transfer of Pacific abalone to the south for culture and the north–south relay within culture patterns in China, as well as the exchange of juvenile abalone between hatcheries, has eliminated the geographical barriers controlling populations to an extent and promoted genetic exchange among populations, and later these populations produced genetic differentiation after a long period of reproduction. These factors have led to a degree of genetic differentiation among diverse Pacific abalone populations and the inconsistency in the relationship between geographic distance and genetic distance [4,47]. In this study, both the NH and DQ Pacific abalone populations lived in the natural waters of the Changshan Archipelago and had the lowest degree of genetic differentiation and the smallest genetic distance, indicating that the degree of genetic differentiation between populations was low and that the genetic relationship was relatively close. The southern cultured population ZZ and northern cultured population RC had the highest degree of genetic differentiation (F_st_ = 0.1334) and the largest genetic distance (0.4167). The main reasons for the higher degree of genetic differentiation among cultured populations may be that a small number of effective parents was selected in the hatcheries during selective breeding [50,51,52], there were different breeding conditions, and long-term artificial culture caused genetic drift and changed the genetic composition.

Hence, the continued prohibition of the introduction of exotic Pacific abalone populations into the waters of the Changshan Archipelago for bottom sowing culture and the use of native juveniles as much as possible for raft cultivation can prevent the escape of exotic populations, which can prevent changes to the genetic composition of Daqin Island wild populations. In addition, Pacific abalone originating from artificially assisted wild population breeding programs can be transferred to the natural waters of the Changshan Archipelago for proliferation and release to restore the wild resources of Pacific abalone.

## 5. Conclusions

In conclusion, thirteen polymorphic EST-SSR loci were developed based on the hemolymph transcriptome data of Pacific abalone and were used to reveal the genetic diversity and genetic structure of six different Pacific abalone populations. The current genetic diversity of different populations is still at a high level, especially in the populations of the Changshan Archipelago, which is the origin of Pacific abalone. Moreover, there was a higher degree of genetic differentiation between the southern cultured population ZZ and the northern cultured populations RC and DL under different breeding conditions. This study enables a better understanding of the genetic diversity and structure of current Pacific abalone populations and provides valuable information for the management and restoration of abalone germplasm resources.

## Figures and Tables

**Table 1:** Sampling information of *H*. *discus hannai.*

Population	Longitude	Latitude	Sampling Date	Sample Size
ZZ	117°51′57.72″	24°7′58.81″	6 November 2020	45
DL	121°31′19″	38°52′26.43″	23 August 2020	50
RC	122°33′37.27″	37°8′40.55″	4 November 2020	39
TJ	120°45′42.87″	38°9′29.19″	23 August 2020	50
DQ	120°50′5.05″	38°17′54.6″	13 March 2021	39
NH	120°54′51.41″	38°20′59.71″	23 August 2020	36

**Table 2:** Description of basic information about the SSR locus in *H. discus hannai.*

Searching Item	Number
total number of sequences examined:	80,032
total size of examined sequences (bp):	1,865,475,499
total number of identified SSRs:	415,996
number of SSR containing sequences:	36,665
number of sequences containing more than one SSR:	22,605
number of SSRs present in compound formation:	102,835
mononucleotide:	82,256 (19.77%)
dinucleotide:	160,881 (38.67%)
trinucleotide:	62,014 (14.91%)
tetranucleotide:	100,776 (24.23%)
pentanucleotide:	7816 (1.88%)
hexanucleotide:	2253 (0.54%)

**Table 3:** Sequences of primers for 13 polymorphic EST-SSR of *H. discus hannai.*

Loci	Primer Sequence	Annealing Temperature	Repeat Motif	Fluorescent Labeling
WXZ-1	F: GACAGGTCAGCCGAAATTGC R: CCAAATGCCTGTGAATGCCC	55 °C	(CAA)13	ROX
WXZ-2	F: TGTGACGGTGACCCTTTGTC R: TCTGAGTGTATTTGGTCCGCA	55 °C	(CAT)12	TAMRA
WXZ-3	F: GGAACCAAACCCAGGTGCTA R: AGTCAAACCCGGAATGCCAG	55 °C	(CTG)6	ROX
WXZ-4	F: TGGGAGGGATGTACCGCATA R: GGTTTGCCCTCTCCAGACTC	55 °C	(GCA)13	TAMRA
WXZ-5	F: AAGGAGGCGACTATTGCACC R: TGTTTCCACGTCTGCCAGTT	55 °C	(CACAC)10	HEX
WXZ-6	F: GGATTGAGGGTTGGGGGATG R: CTTGGCCTGCAAGCTGATTG	55 °C	(CTA)9	TAMRA
WXZ-7	F: GGAAAGTGCCAAGTGGTGTG R: TACCCTACTGCCCCACCATC	55 °C	(GAT)15	ROX
WXZ-8	F: GATCTACCAGAGCCCACTGC R: TGACATCGTGAGCTTCGACC	55 °C	(CAT)9	FAM
WXZ-9	F: CAAAACCACAAGCATGGCGA R: TGCGTTCGACCTGTCAGAAG	55 °C	(ATC)17	FAM
WXZ-10	F: TCACTGATGTCGATTTTGCGC R: TCCACCTTTGGCGTCAGTTT	55 °C	(ATT)14	FAM
WXZ-11	F: ACCTCGCATCAACAATCGGT R: CCCCAATCCTGCTTTTTGGC	55 °C	(TGT)8	FAM
WXZ-12	F: TGACCACAGTGACATCGACA R: CCTGTCATATAACGCGGGGG	55 °C	(GACA)8	HEX
WXZ-13	F: CGCCATTGCAACTGCGTTAT R: AGTGGAAGCGACACGACAAT	55 °C	(GAACC)8	HEX

**Table 4:** The main genetic parameters of six populations in *H. discus hannai.*

Loci	Parameters	Population					
		DL	NH	DQ	TJ	RC	ZZ
WXZ-1	Na	12	11	14	13	9	10
	Ne	4.8868	6.4815	9.2862	5.6761	5.6860	4.2883
	PIC	0.7800	0.8279	0.8827	0.8080	0.8010	0.7430
	Ho	0.8478	0.6857	0.7632	0.8367	1.0000	0.8537
	He	0.8041	0.8580	0.9042	0.8323	0.8348	0.7763
	F_is_	−0.0660	0.1892	0.1447 *	−0.0157	−0.2134	−0.1133
	F null	0.0000	0.0837	0.0714	0.0820	0.0000	0.0000
WXZ-2	Na	6	6	6	6	6	6
	Ne	5.3570	3.7405	4.2250	4.7357	4.8907	3.9918
	PIC	0.7868	0.6946	0.7339	0.7590	0.7653	0.7194
	Ho	0.9130	0.7143	0.7692	0.8571	0.7949	0.6818
	He	0.8223	0.7433	0.7732	0.7970	0.8059	0.7581
	F_is_	−0.1226	0.0251	−0.0078	−0.0866	0.0008	0.0903
	F null	0.0438	0.1205	0.1284	0.0589	0.1099	0.1783
WXZ-3	Na	12	14	18	17	10	11
	Ne	7.6701	5.1042	6.5786	10.5771	6.9294	8.2915
	PIC	0.8560	0.7861	0.8371	0.8982	0.8397	0.8680
	Ho	0.9362	0.8857	0.9211	0.9796	0.8462	0.8605
	He	0.8790	0.8157	0.8593	0.9148	0.8668	0.8897
	F_is_	−0.0765	−0.1015	−0.0862	−0.0819	0.0111	0.0215 *
	F null	0.0000	0.0019	0.0126	0.0000	0.0000	0.0766
WXZ-4	Na	7	9	9	9	5	5
	Ne	3.4195	2.3330	2.0599	3.9925	2.9194	1.5624
	PIC	0.6761	0.5478	0.4982	0.7269	0.6081	0.3431
	Ho	0.7021	0.4118	0.2368	0.4348	0.5641	0.3333
	He	0.7152	0.5799	0.5214	0.7578	0.6660	0.3650
	F_is_	−0.0077	0.2793 *	0.5397 *	0.4199 *	0.1420	0.0740
	F null	0.1031	0.1800	0.2158	0.2698	0.0412	0.0477
WXZ-5	Na	11	12	16	13	9	11
	Ne	7.6835	6.5860	8.1120	7.1373	5.8500	5.8401
	PIC	0.8564	0.8322	0.8649	0.8451	0.8065	0.8109
	Ho	0.8936	0.8857	0.8462	0.8298	0.9231	0.7500
	He	0.8792	0.8605	0.8881	0.8691	0.8398	0.8383
	F_is_	−0.0273	−0.0443	0.0349	0.0350	−0.1134	0.0950
	F null	0.0000	0.0234	0.0000	0.0024	0.0000	0.0447
WXZ-6	Na	8	8	10	11	9	7
	Ne	6.5295	5.2326	6.0504	5.9297	5.0417	4.7964
	PIC	0.8281	0.7835	0.8149	0.8147	0.7773	0.7646
	Ho	0.5227	0.2667	0.4138	0.5778	0.4848	0.4884
	He	0.8566	0.8226	0.8494	0.8407	0.8140	0.8008
	F_is_	0.3827 *	0.6073 *	0.5043 *	0.3050 *	0.3952 *	0.3830 *
	F null	0.1865	0.2985	0.2541	0.1742	0.2247	0.3135
WXZ-7	Na	7	11	12	10	8	10
	Ne	3.2891	4.3286	4.1786	4.5259	4.5134	6.5739
	PIC	0.6478	0.7431	0.7327	0.7500	0.7493	0.8309
	Ho	0.7083	0.6857	0.7436	0.7959	0.8974	0.8182
	He	0.7033	0.7801	0.7706	0.7871	0.7885	0.8576
	F_is_	−0.0178	0.1083	0.0225	−0.0217	−0.1529	0.0350 *
	F null	0.0000	0.0315	0.0042	0.0000	0.0000	0.0695
WXZ-8	Na	8	10	9	9	7	9
	Ne	5.4468	5.3728	4.5882	5.8561	5.0226	2.9969
	PIC	0.7929	0.7897	0.7496	0.8078	0.7765	0.6437
	Ho	0.9167	0.8000	0.7949	0.8571	0.7895	0.7045
	He	0.8250	0.8257	0.7922	0.8378	0.8116	0.6740
	F_is_	−0.1228	0.0171	−0.0164	−0.0337	0.0143	−0.0574
	F null	0.0000	0.0151	0.0000	0.0000	0.0000	0.0397
WXZ-9	Na	11	16	13	10	8	8
	Ne	5.9689	7.8108	7.6432	5.8418	5.2813	4.8851
	PIC	0.8126	0.8611	0.8562	0.8077	0.7847	0.7692
	Ho	0.8958	0.8824	0.8974	0.9184	0.8205	0.8372
	He	0.8412	0.8850	0.8805	0.8374	0.8212	0.8047
	F_is_	−0.0761	−0.0119	−0.0325	−0.1080	−0.0122	−0.0527
	F null	0.0000	0.0198	0.0000	0.0000	0.0000	0.0000
WXZ-10	Na	8	11	9	11	8	8
	Ne	4.1143	5.7110	5.0578	5.7235	6.4586	4.4879
	PIC	0.7221	0.8057	0.7788	0.8026	0.8266	0.7471
	Ho	0.7708	0.7429	0.8421	0.7755	0.7949	0.6279
	He	0.7649	0.8369	0.8130	0.8338	0.8561	0.7863
	F_is_	−0.0183	0.0995	−0.0496	0.0603	0.0595	0.1921 *
	F null	0.0000	0.0591	0.0000	0.0605	0.0238	0.2029
WXZ-11	Na	12	11	9	13	9	9
	Ne	5.9211	4.6402	3.0022	5.7855	3.3065	3.0156
	PIC	0.8127	0.7577	0.6277	0.8085	0.6756	0.6346
	Ho	0.8667	0.8000	0.8108	0.7959	0.6154	0.6818
	He	0.8404	0.7959	0.6760	0.8357	0.7066	0.6761
	F_is_	−0.0428	−0.0198	−0.2158	0.0378	0.1178	−0.0201
	F null	0.0084	0.0000	0.0000	0.0485	0.0455	0.0239
WXZ-12	Na	8	8	9	8	7	10
	Ne	2.9425	5.1797	5.7723	5.5314	3.8073	5.3672
	PIC	0.6315	0.7808	0.8046	0.7955	0.7061	0.7900
	Ho	0.5625	0.8286	0.8462	0.7955	0.8205	0.9535
	He	0.6671	0.8186	0.8375	0.8286	0.7469	0.8233
	F_is_	0.1479	−0.0268	−0.0235	0.0290	−0.1128	−0.1718
	F null	0.1194	0.0000	0.0286	0.1230	0.0095	0.0000
WXZ-13	Na	14	13	14	13	10	10
	Ne	5.4677	10.3814	9.5063	5.2196	6.6419	5.7705
	PIC	0.8027	0.8955	0.8858	0.7908	0.8319	0.8044
	Ho	0.5870	0.6857	0.6154	0.5510	0.6410	0.7727
	He	0.8261	0.9168	0.9064	0.8167	0.8605	0.8362
	F_is_	0.2817 *	0.2412 *	0.3123 *	0.3184 *	0.2454 *	0.0653 *
	F null	0.1307	0.1188	0.1656	0.1381	0.1039	0.0406
Average	Na	9.5385	10.7692	11.3846	11.0000	8.0769	8.7692
	Ne	5.2844	5.6079	5.8508	5.8871	5.1038	4.7590
	Ho	0.7787	0.7135	0.7308	0.7696	0.7686	0.7203
	He	0.8019	0.8107	0.8055	0.8299	0.8014	0.7605

Wide significance levels were applied using the sequential Bonferroni technique (k = 13); * indicates significant deviation from the Hardy–Weinberg equilibrium (*p* < 0.004).

**Table 5:** The F_st_ value (below diagonal) and genetic distance (above diagonal) of six populations in *H. discus hannai.*

Population	DL	ZZ	NH	DQ	RC	TJ
DL		0.2937	0.2718	0.2399	0.219	0.1133
ZZ	0.0992 **		0.229	0.1826	0.4167	0.2533
NH	0.0759 **	0.0728 **		0.0511	0.3175	0.2099
DQ	0.0685 **	0.0608 **	0		0.3131	0.1766
RC	0.0661 **	0.1334 **	0.1007 **	0.0869 **		0.2688
TJ	0.0271 **	0.0783 **	0.0514 **	0.0439 **	0.0736 **	

Note: ** indicates that the F_st_ reaches a significant level at *p* < 0.01.

**Table 6:** Analysis of molecular variance (AMOVA) of 6 populations in *H. discus hannai.*

Groups	Source of Variation	Degree of Freedom	Sum of Squares	Variance Component	Source of Variation	*p*-Value
6 populations	Among populations	5	119.157	0.214	4%	
	Among individuals	248	1427.921	0.489	9%	
	Within individuals	254	1214.000	4.780	87%	
	Total	507	2761.079	5.483	100%	0.001
6 populations, divided into wild group and cultured group	Among populations	1	14.957	0.067	1%	
	Among individuals	252	1532.121	0.650	12%	
	Within individuals	254	1214.000	4.780	87%	
	Total	507	2761.079	5.497	100%	0.001

## Data Availability

Data are contained within the article.
